# Effects of experimentally induced lumbar nociception on trunk motor control in the rat during locomotion

**DOI:** 10.1007/s00221-025-07041-8

**Published:** 2025-04-28

**Authors:** Fangxin Xiao, Wendy Noort, Juliette Lévénez, Jia Han, Jaap H. van Dieën, Huub Maas

**Affiliations:** 1 Department of Human Movement Sciences, Faculty of Behavioural and Movement Sciences, Vrije Universiteit Amsterdam, Amsterdam Movement Sciences, Van der Boechorststraat 7, Amsterdam, 1081 BT The Netherlands; 2 School of Exercise and Health, Shanghai University of Sport, Shanghai, China; 3 College of Rehabilitation Sciences, Shanghai University of Medicine and Health Sciences, Shanghai, China; 4 Faculty of Health, Arts and Design, Swinburne University of Technology, Hawthorn, VIC Australia

**Keywords:** Nociception, Neuromuscular control, Electromyography (EMG), Locomotion, Gait, Animal model

## Abstract

Nociception resulting in pain perception might be one of the factors contributing to the motor control changes in people with low-back pain. However, limited evidence exists regarding the effects of acute pain on trunk motor control during locomotion. This study aimed to evaluate the effects of hypertonic saline induced nociception on trunk movement and back muscle activity during locomotion in a rat model. Spine and pelvis kinematics, EMG signals from bilateral multifidus (MF) and medial longissimus (ML) muscles of the rats were collected during treadmill locomotion before and after hypertonic saline (5.8%) injection into the MF. We found that both the locomotion and EMG patterns remained unchanged after hypertonic saline injection. No significant changes were found in stride duration, pelvic, lumbar and spine angle changes, variability, or movement asymmetry. The overall EMG activation patterns and intermuscular coordination remained unchanged after hypertonic saline injection and there was synchronized activation of bilateral MF muscles with two peaks per stride cycle, and alternating activation of left and right ML. The only significant effects of hypertonic saline injection were the decrease in the normalized peak amplitude of the left MF and EMG variability in right ML, no effects were detected in other EMG outcomes or muscles. These results suggest that the changes in EMG activity reflect localized neuromuscular response to nociception rather than broader alterations in control of locomotion.

## Introduction

Differences in motor control have been observed between individuals with and without low-back pain (LBP), but findings on changes in trunk muscle activity are inconsistent (Hodges and Tucker 2011; van Dieen et al. 2003). Studies reported increased, decreased, or unchanged trunk muscle activity in people with LBP (van Dieen et al. 2003), with evidence suggesting inhibition of deep muscles (e.g. multifidus) and increased activation of superficial muscles (e.g. longissimus) (Devecchi et al. 2023; van Dieen et al. 2003, 2019). It has been proposed that nociception resulting in pain perception is one of the mechanisms contributing to these changes, leading to adaptations to protect the body region from further pain (van Dieen et al. 2019). Such adaptations include redistribution of activity within and between muscles (Hodges and Tucker 2011), delayed responses to perturbations (Prins et al. 2018), and protective gait strategies such as increased trunk stiffness (Hodges et al. 2009) and rigid pelvis-trunk coordination (Lamoth et al. 2002). Given that clinical pain cannot be studied separately from psychological factors, such as fear of movement, that may also affect muscle activity, the physiological responses to acute pain on trunk motor control during locomotion are still unclear.

Experimental LBP models using intramuscular hypertonic saline (4–6% (Svendsen et al. 2005) injections are widely employed to investigate the effects of pain on motor control (Hodges et al. 2003, 2013; Tsao et al. 2011). Hypertonic saline induces acute, localized, and reproducible pain through activation of nociceptors (Graven-Nielsen et al. 1997a, b), without causing tissue damage (Svendsen et al. 2005) or affecting muscle fibre electrophysiological properties (Farina et al. 2005; Qerama et al. 2005). However, in humans, psychological responses to experimentally induced pain may still vary widely and as such cause different changes in muscle activity. Most of the experimental LBP studies performed in humans have focused on static postures or controlled movements, with limited evidence on effects of acute pain on neural control of the lumbar muscles during functional tasks such as locomotion (Devecchi et al. 2023; van Dieen et al. 2003). Previous studies showed that hypertonic saline injection into the lumbar erector spinae muscle led to an overall increased mean EMG amplitude of the erector spinae muscle, especially during the ipsilateral swing phase, but decreased EMG peak and mean amplitude during the double stance phase (Arendt-Nielsen et al. 1996; Lamoth et al. 2004). Interestingly, these changes occurred without affecting the gait cycle duration (Arendt-Nielsen et al. 1996) or trunk kinematics (Lamoth et al. 2004).

In animal models, intramuscular hypertonic saline injection has also been used frequently to induce experimental muscle pain (Capra and Ro 2004). It activates group III and IV afferents (Hoheisel et al. 2005; Paintal 1960), triggering pain responses lasting for 2–5 min (Paintal 1960). Studies in lightly anesthetized rats demonstrated that hypertonic saline (100 µl) intramuscular injection into the masseter and gastrocnemius muscles reliably induces pain responses (Bagues et al. 2014; Ro et al. 2003). Hypertonic saline (50 µl) injection into the L4-L5 multifidus muscle of the rat has been demonstrated to excite the dorsal horn neurons at a short latency (Taguchi et al. 2008), confirming its effectiveness in eliciting nociceptive signaling. Therefore, hypertonic saline injection provides a controlled means to study the effects of nociception on muscle activation. This study aimed to evaluate the effects of hypertonic saline induced nociception on trunk movement and back muscle activity during locomotion in a rat model.

## Method

### Animals

All experimental procedures were in accordance with the Dutch law on animal research in full agreement with the Directive 2010/63/EU and approved by the Netherlands Central Commission for Animal Experiments (Permit Number AVD11200202115388). Local approval and supervision were provided by the Animal Welfare Body at the Vrije Universiteit Amsterdam.

Twelve adult male Wistar rats (*Rattus norvegicus domestica*, 330 ± 34 gram prior to surgery) were used in this study. Only male rats were used because of the sex difference in pain response reported in literature (Cairns et al. 2001; Capra and Ro 2004). Upon arrival (9 weeks of age), rats were housed in pairs under a 12-h light/dark cycle. After EMG electrode implantation surgery (12 weeks of age), rats were housed in the same cage but separated by a cage divider, allowing to see and smell each other. Rats were allowed to move freely in the cage with access to food and water *ad libitum*.

### Study preregistration

This study was preregistered at PreclinicalTrials.eu prior to conducting the research (registration number PCTE0000367). The preregistration adheres to the disclosure requirements of the institutional registry.

### Experimental protocol

**Fig. 1:**
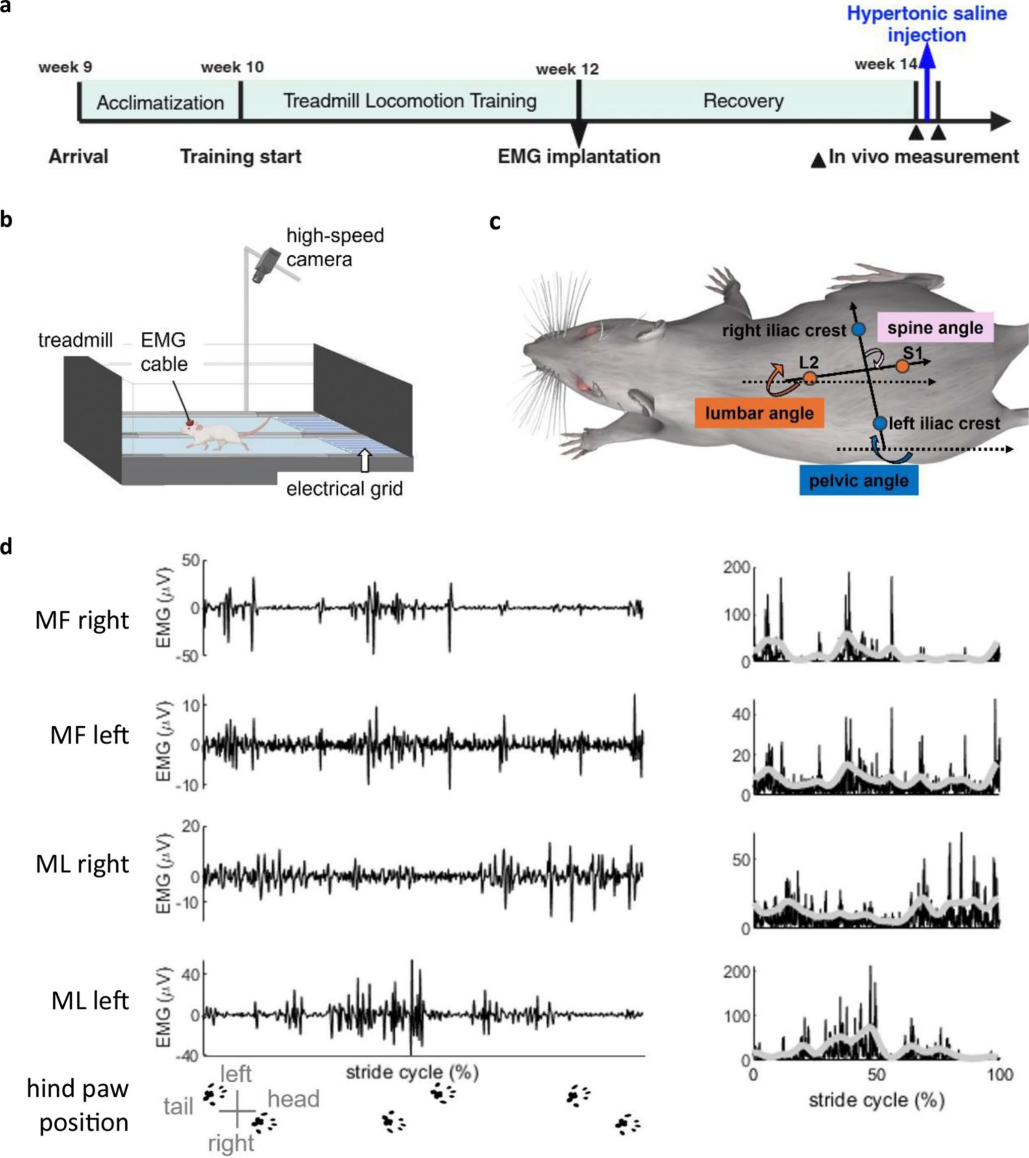
Overview of experimental protocol and data collection procedures. **(a)** Experimental timeline. * age of the rat.**(b)** in vivo measurement setup. **(c)** Illustration of motion tracking markers and the convention of joint angles. **(d) (left)** Representative band-pass filtered electromyograms (EMG) and **(right)** rectified (black line) and filtered (light grey line) EMG signals of bilateral multifidus (MF) and medial longissimus (ML) muscles from one stride cycle of the rat trotting on the treadmill at 0.5 m/s before hypertonic saline injection (rat S10). Bottom row shows the hind paw position of the rat during locomotion

After one week of acclimatization, the rats were trained to run on a treadmill (Exer 3/6, Columbus Europe Instruments, Dublin, Ireland) for two weeks, then four pairs of Teflon-insulated fine-wire electromyography (EMG) electrodes (7SS-1T, Science Products, Hofheim, Germany) were implanted into the multifidus muscle (MF) and medial longissimus muscle (ML) between L4 and L5 vertebra bilaterally. After two weeks of recovery, in vivo measurements were performed to collect MF and ML EMG signals, as well as spine and pelvis kinematics, before and after hypertonic saline (5.8%) injection into the MF muscle (Fig. 1a).

### Treadmill locomotion training

Prior to implantation of the EMG electrodes, rats were trained to run on a motor-driven treadmill for two weeks on a daily basis. Each training lasted for 10–20 min with increasing speed up to 0.6 m/s and consisted of several 2–3 min running sessions. Food reward followed by approximately 5 min rest was provided upon finishing each running session. To promote running, an electrical grid located behind the treadmill delivered a mild electrical stimulus (repetition rate: 2 Hz, stimulus current: max 1 mA, stimulus duration: 200 ms) once the animal stepped on the platform behind the treadmill. The electrical stimulus was automatically switched off once the rat stepped on the grid for a third time. In addition, before touching the grid, the rats would first come into contact with a small object hanging in front of the grid. This object served as a warning signal to avoid stepping on the platform.

### Surgical procedures for EMG electrodes implantation

#### Animal Preparation

Carprofen (3 mg/kg, Rimadyl^®^, Zoetis B.V., Capelle a/d Ijssel, The Netherlands) was administrated subcutaneously 12 h before surgery. Both carprofen (3 mg/kg) and buprenorphine (0.02 mg/kg, Buprecare^®^, Ecuphar NV, Oostkamp, Belgium) were administrated subcutaneously 30–60 min before surgery. The rats were anaesthetized by isoflurane (induction in a box: 3–5%, maintenance via nose cone: 1–2%), then mounted in a stereotaxic frame (David Kopf Instruments, Tujunga, CA, USA) and placed on a heating pad. Eye ointment was applied during surgery to prevent dehydration. The local anesthetic Ropivacaine (2 mg/kg, Fresenius Kabi Norge AS, Halden, Norway) was applied several minutes before incision. Hind paw pain reflex, breathing rate, and rectal temperature were monitored throughout the surgery.

#### EMG connector

The EMG connector was mounted on the head of the animal. A skin incision (~ 1 cm) was made on top of the skull, and several drops of lidocaine with HCl (1%, 10 mg/ml, B. Braun, Melsungen, Germany) were locally applied to prevent excessive bleeding. Another skin incision (~ 1–2 cm) was made over the lower lumbar spine, then the electrode wires were threaded subcutaneously from the head down to the dorsal lumbar region. After exposing the skull, four stainless steel screws were placed on the skull, two in front of the bregma and two behind. The EMG connector was placed between the four screws, and anchored to the skull with dental cement (RelyX Unicem2 Automix, 3 M ESPE, Germany) to encapsulate the bottom part of the connector and all the screws. The skin was closed with sutures (4 − 0, ETHIBOND EXCEL, non-absorbable, ETHICON).

#### Implantation EMG electrodes

After securing the connector on the head, four pairs of EMG electrodes were implanted bilaterally into MF and ML between the L4 and L5 vertebral levels, using procedures described in our previous study (Bernabei et al. 2017) and Tysseling et al. (Tysseling et al. 2010). Briefly, a 27-gauge needle bent at 90° was inserted into the muscle belly, then the electrode wires were threaded into the needle and the needle was withdrawn, the two distal end of the electrode wires were then tied in a knot to secure the electrode within the muscle and the superfluous wire was trimmed. The EMG electrodes were implanted approximately 2 mm deep and approximately 1 mm apart, electrode placement was verified by electrical stimulation through the implanted wires. A pair of reference electrodes was inserted underneath the skin, in the region above the gluteus maximus muscle. Electrodes placement was further verified by dissecting the implanted muscles after termination of the animals. The skin was closed with sutures (5 − 0, Vicryl, absorbable, ETHICON). At completion of the surgery, 4 ml lactated ringer solution was injected subcutaneously. Then the rats were transferred back to a recovery cage placed on a heating pad and monitored for recovery.

### Intramuscular hypertonic saline injection to induce nociception

Literature (Bagues et al. 2014; Hoheisel et al. 2005; Ro et al. 2003; Taguchi et al. 2008) and a pilot study (unpublished data) demonstrated that injection of 100 µl hypertonic saline into the rat’s MF muscle caused mild-to-maximum moderate pain responses based on the Rat Grimace Scale (score ranged: 0.5-1) (Leung et al. 2016; Miller et al. 2016). Therefore, in this study nociception was induced by injecting 100 µl hypertonic saline (5.8%) solution randomly into either the left or right MF muscle between the L4 and L5 vertebral levels using an insulin syringe according to the method described by Taguchi et al. (Taguchi et al. 2008). In brief, while the rats were under ultra-short isoflurane anesthesia, the needle was advanced into the muscle beside the spinous process until it contacted the bone of the vertebral arches. Then the needle was withdrawn for 1 mm to release the saline solution into the muscle.

### In vivo measurement and data analysis

Before and after hypertonic saline injection, in vivo measurements were performed to collect locomotion data and EMG signals, while the rats were trotting on the treadmill at a fixed speed of 0.5 m/s (Fig. 1b). Kinematics data and EMG signals were synchronized by an electronic trigger pulse to the controller (Digital Sonomicrometer, Sonometrics, London, ON, Canada).

#### Kinematics

Two-dimensional videos of treadmill locomotion were recorded using a high-speed camera (A602f, Basler, Ahrensburg, Germany) placed above the treadmill (Fig. 1b). Videos were sampled at 200 frames/s and recorded at a computer hard drive with custom software (Labview, National Instruments, Austin, TX). Skin markers were placed on the rat’s spine at the L2 and S1 spinous processes, as well as on the pelvis at the left and right iliac crests (Fig. 1c).

#### Electromyography

EMG signals of MF and ML were amplified (1250×, common-mode rejection ratio > 100 dB), filtered (10–1175 Hz) and sampled (3123 Hz). Band-pass digital filters (100–1000 Hz, 3rd order zero-lag Butterworth) were applied for signal processing to remove movement artifacts and treadmill noise. The EMG linear envelope was computed as the magnitude of the discrete-time analytic signal calculated by the Hilbert transform and low-pass filtered (25 Hz, 2nd -order zero-lag Butterworth).

#### Data analysis

Videos within 5 min (Paintal 1960) after hypertonic saline injection were analyzed in DeepLabCut (Nath et al. 2019) to obtain the time series of the segmental angle data between the tracked markers (lumbar angle: marker L2 to S1; pelvic angle: marker left iliac crest to right iliac crest). As shown in Fig. 1c, the lumbar and pelvic angles were measured with reference to a horizontal line with the positive direction to the right. Extreme values in the segmental angle data (pelvic angle data beyond the range of 250–300 degree, lumbar angle data beyond the ranges of 0–30 degree and 330–360 degree) were removed and interpolated using the Piecewise Cubic Hermite Interpolating Polynomial method, then the angle data were low-pass filtered (5 Hz, 3rd order zero-lag Butterworth). The pelvic angle was used to separate stride cycles, and the start of the stride cycle was defined as the video frame in which the pelvic angle was minimal. Stride cycles of trotting at constant speed (cycle duration range: 0.2–0.4 s) were used for data analysis, galloping gaits and strides with forward-backward acceleration or left-right swing on the belt were excluded from analysis. Subsequently, the angle data were normalized to the stride cycle duration and interpolated to 100 data points. EMG data were also time-normalized to 100 time samples per stride. For each rat, a mean across stride cycles from the same measurement session was calculated, and the amplitude of the EMG envelope for each normalized time-point was normalized to the maximum value of the mean EMG recorded during baseline.

For each rat, changes in lumbar and pelvic angle over the stride were calculated based on the time-normalized segmental angle data. The offset of the angle data possibly caused by asymmetry in marker placement was removed by subtracting the mean of the time series. The spine angle was defined as the relative angle between the lumbar and pelvic angles. The timing of the peak pelvic angle was described as the percentage of the stride cycle at maximum pelvic angle. Variability of angle changes was expressed as the mean of the standard deviations across stride cycles for each time point. Movement asymmetry was calculated as the standard deviation of the differences between corresponding points in the first and second half of the spine angle curve, divided by half of the peak-to-peak difference of the curve. The peak and minimum EMG amplitude were defined as the maximum and minimum value in the normalized EMG envelope, respectively. The average EMG activity was calculated as the mean of the normalized EMG envelope. The mean of the standard deviations across stride cycles was used to indicate the variability of EMG and was calculated as described above for the kinematics. Each variable was averaged within rats.

#### Statistical analysis

EMG data from specific channels were excluded when there was a bad signal-to-noise ratio, electrode malfunction or inappropriate placement of the electrodes. Paired t-tests were performed to assess the effects of nociception on kinematics (pelvic/lumbar/spine angle change and variability, movement asymmetry), MF and ML EMG activity (peak amplitude, minimum amplitude, mean amplitude, variability). All statistical analysis were performed using MATLAB R2021a (MathWorks, Inc., Natick, MA, United States). Results were considered significant when *p* < 0.05, Cohen’s D was calculated as effect size. Data are presented as mean (SD).

The adopted statistical analysis deviated from the preregistered analysis plan (two-way repeated measures ANOVA). Two interventions have been preregistered, but only one intervention (hypertonic saline injection to induce nociception) is reported here. The other intervention (intervertebral disc injury, which was performed after the hypertonic saline injection) and the interaction between these two interventions will be reported later.

## Results

Body weight of the rats at time of termination was 373 ± 33 gram. Data from one rat were excluded from data analysis due to poor pelvic marker recognition and malfunctioning EMG electrodes. In addition, EMG data were excluded from specific muscles, because of low signal-to-noise ratio, improper electrode placement, or malfunctioning electrodes, including one rat for right MF, three rats for left MF, and one rat for left ML. The time points of EMG signals recorded after hypertonic saline injection was 2.3 ± 1.1 min (range:1–5 min).

**Fig. 2:**
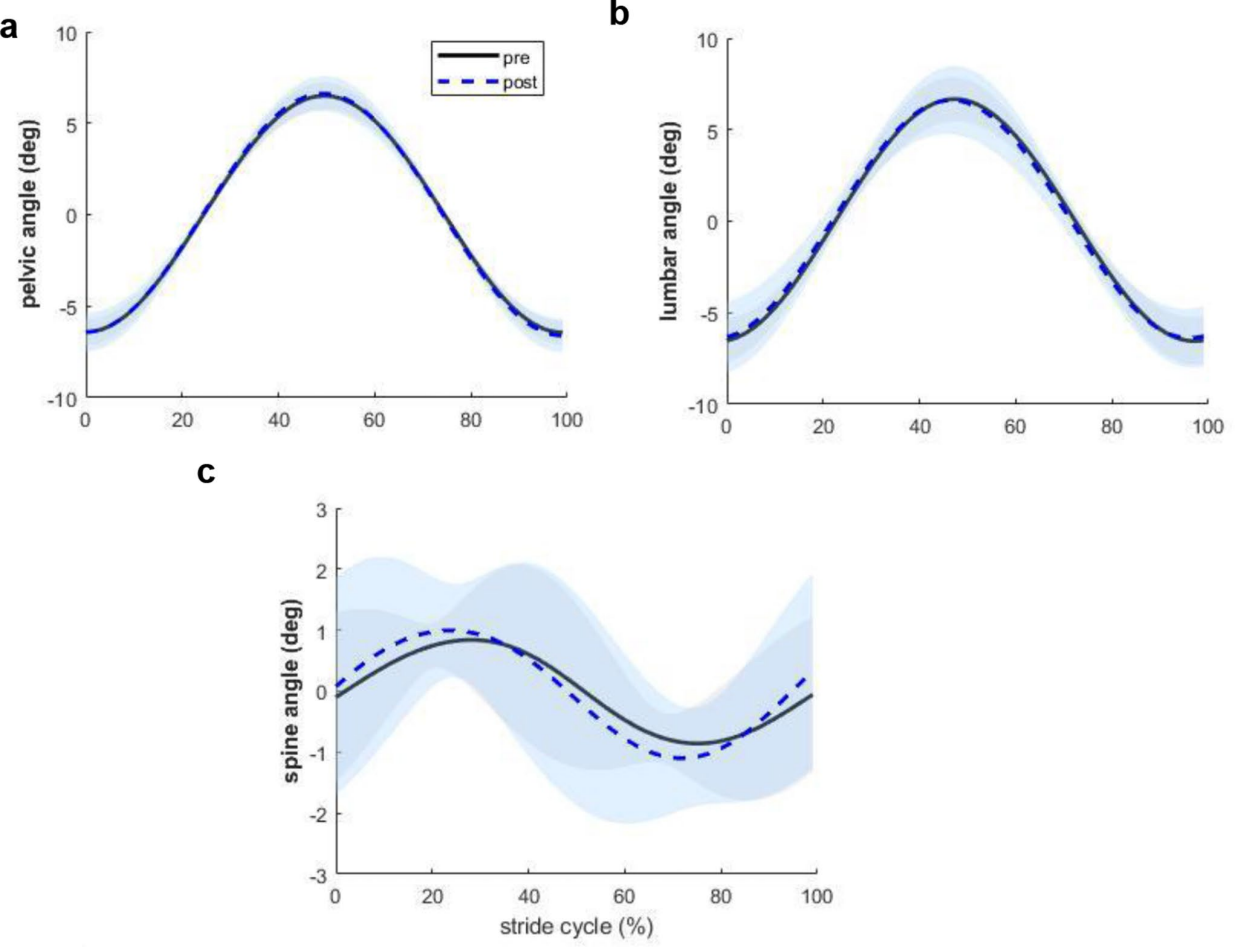
Joint angle changes during locomotion pre- (black solid line) and post- (blue dashed line) hypertonic tonic saline injection. **(a)** pelvic, **(b)** lumbar, **(c)** spine. Joint angle data were averaged across 11 rats and normalized to stride cycle duration. Shaded area represents mean ± 1SD. Treadmill speed was at 0.5 m/s in both conditions

Across strides and rats, a consistent locomotion pattern was observed both before and after hypertonic saline injection. There was no change in either stride cycle duration or the timing of peak pelvic angle with nociception (Table 1). The peak pelvic angle occurred at 50% of the stride cycle (Fig. 2a), so did the peak lumbar angle (Fig. 2b), and the spine angle changes over the stride cycle resembled a sinusoidal curve (Fig. 2c). No effects of nociception were detected on any of the kinematic outcomes (*p > 0.05*, Table 2).

**Table 1:** Kinematic outcomes pre- and post-hypertonic saline injection

	pre	post
cycle duration (sec)	0.32 (0.02)	0.31 (0.02)
pelvic angle change (deg)	13.07 (1.39)	13.34 (1.84)
pelvic angle variability	1.33 (0.22)	1.36 (0.17)
timing of peak pelvic angle (%)	50.64 (0.63)	50.17 (0.69)
lumbar angle change (deg)	13.47 (2.33)	13.31 (3.53)
lumbar angle variability	1.90 (0.79)	2.27 (0.98)
spine angle change (deg)	2.94 (1.61)	3.86 (1.83)
spine angle variability	1.54 (0.97)	1.99 (1.16)
movement asymmetry (%)	13.65 (7.61)	12.80 (7.91)

Sample size = 11, data are presented as mean (SD)

**Table 2:** Statistical results for kinematic and EMG outcomes pre- and post-hypertonic saline injection

		t-value	df	*p*-value	95% CI		effect size
					lower	upper	
**kinematic**	cycle duration	1.34	10	0.211	-0.005	0.019	0.40
	pelvic angle change	-0.53	10	0.611	-1.393	0.862	-0.16
	pelvic angle variability	-0.55	10	0.597	-0.162	0.098	-0.16
	timing peak pelvic angle	1.66	10	0.129	-0.161	1.089	0.50
	lumbar angle change	0.16	10	0.874	-2.035	2.355	0.05
	lumbar angle variability	-1.50	10	0.164	-0.925	0.180	-0.45
	spine angle change	-1.72	10	0.116	-2.116	0.272	-0.52
	spine angle variability	-1.45	10	0.178	-1.133	0.240	-0.44
	movement asymmetry	0.29	10	0.781	-0.058	0.075	0.09
**EMG**	MF right	1.30	9	0.227	-0.078	0.287	0.41
peak amplitude	MF left	2.50	7	0.041*	0.012	0.431	0.88
	ML right	1.84	5	0.126	-0.083	0.497	0.75
	ML left	1.53	5	0.186	-0.074	0.291	0.63
minimum amplitude	MF right	0.32	9	0.758	-0.066	0.088	0.10
	MF left	1.40	7	0.203	-0.050	0.196	0.50
	ML right	0.63	5	0.556	-0.045	0.074	0.26
	ML left	0.96	5	0.382	-0.079	0.173	0.39
mean amplitude	MF right	0.06	9	0.954	-0.077	0.081	0.02
	MF left	2.18	7	0.066	-0.011	0.279	0.77
	ML right	2.49	5	0.055	-0.004	0.249	1.02
	ML left	1.69	5	0.152	-0.054	0.260	0.69
variability	MF right	0.49	9	0.638	-0.474	0.774	0.15
	MF left	1.93	7	0.095	-0.113	1.441	0.68
	ML right	3.00	5	0.030*	0.108	2.284	1.23
	ML left	1.66	5	0.158	-0.247	1.549	0.68

MF, multifidus muscle; ML, medial longissimus muscle; df, degree of freedom; CI, confidence interval; effect size, Cohen’s D; *significant difference

**Fig. 3:**
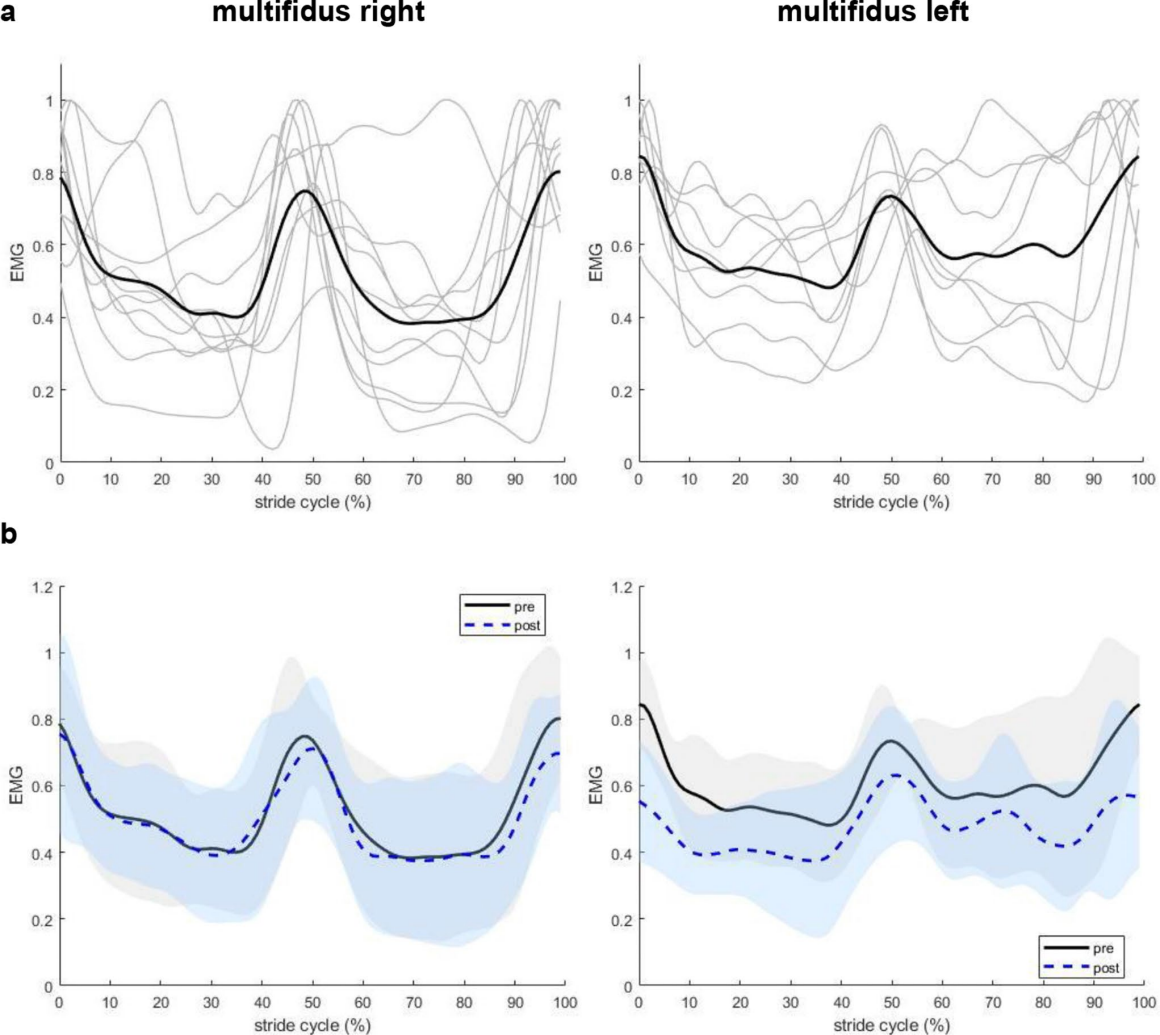
Filtered rectified EMG signal of right (*n* = 10) and left (*n* = 8) multifidus muscles during locomotion. EMG data were normalized to the stride cycle duration and peak amplitude measured before hypertonic saline injection. **(a)** Data of individual rats (thin grey lines) and averaged across rats (thick black line) before hypertonic saline injection. **(b)** Mean ± SD are compared pre- (black solid lines) and post- (blue dashed lines) hypertonic saline injection. Shaded area represents mean ± 1SD. Treadmill speed was at 0.5 m/s in both conditions

**Fig. 4:**
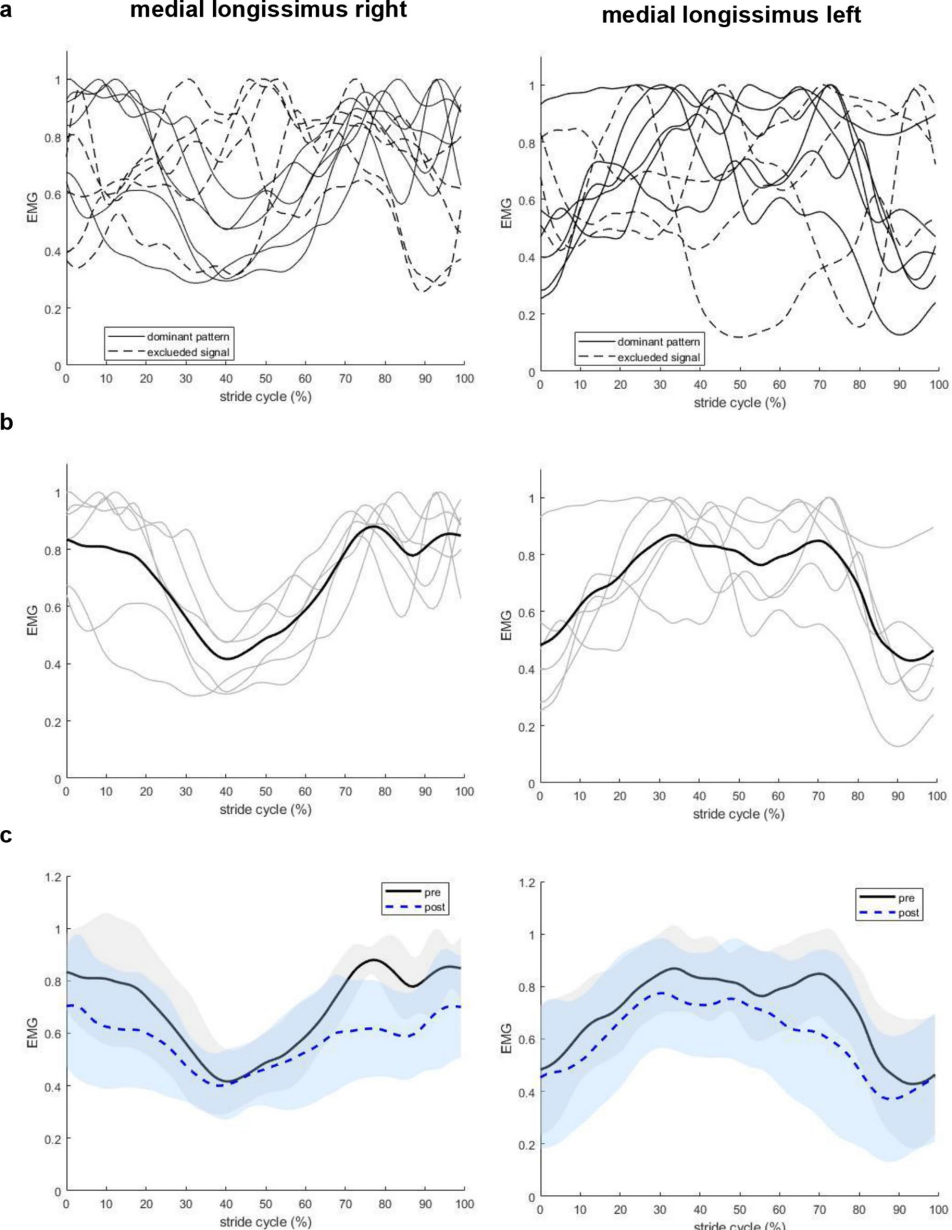
Filtered rectified EMG signal of right and left medial longissimus muscles during locomotion, normalized to the stride cycle duration and peak amplitude measured before hypertonic saline injection. **(a)** Data of right (*n* = 11) and left (*n* = 10) ML muscles for individual rats before hypertonic saline injection. Solid lines represent the baseline pattern observed in ML muscles, dashed lines represent data that were excluded for statistical analysis. **(b)** Dominant pattern of right (*n* = 6) and left (*n* = 6) ML EMG for individual rats (thin grey lines) and averaged across rats (black thick line) before hypertonic saline injection. **(c)** Mean ± SD are compared pre- (black solid lines) and post- (blue dashed lines) hypertonic saline injection. Shaded area represents mean ± 1SD. Treadmill speed was keep at 0.5 m/s at both conditions

EMG patterns did not change after hypertonic saline injection. Both right and left MF showed two peaks per stride cycle, one at the start/end of the stride cycle and another at the middle of the stride cycle. The occurrence of these two peaks corresponds approximately to the stance phase of either the left hind paw (peak at start/end) or right hind paw (peak in the middle), which also aligns with the timing of the minimum and maximum pelvic and lumbar angles. Activation of the left and right MF was synchronized, and the injection of hypertonic saline did not alter this pattern (Fig. 3). High inter-individual variability was observed in ML EMG patterns (Fig. 4a), so the baseline ML EMG patterns (before hypertonic saline injection) were identified based on established descriptions of rat ML EMG pattern during locomotion in the literature (Geisler et al. 1996), and data deviating from the expected pattern were excluded from further analysis (Fig. 4b). An alternating activation of ML between left and right sides was observed (Fig. 4b), as evidenced by an increase in ML EMG activity during the stance phase of the contralateral and swing phase of the ipsilateral hind paw, which was also not affected by nociception (Fig. 4c).

**Fig. 5:**
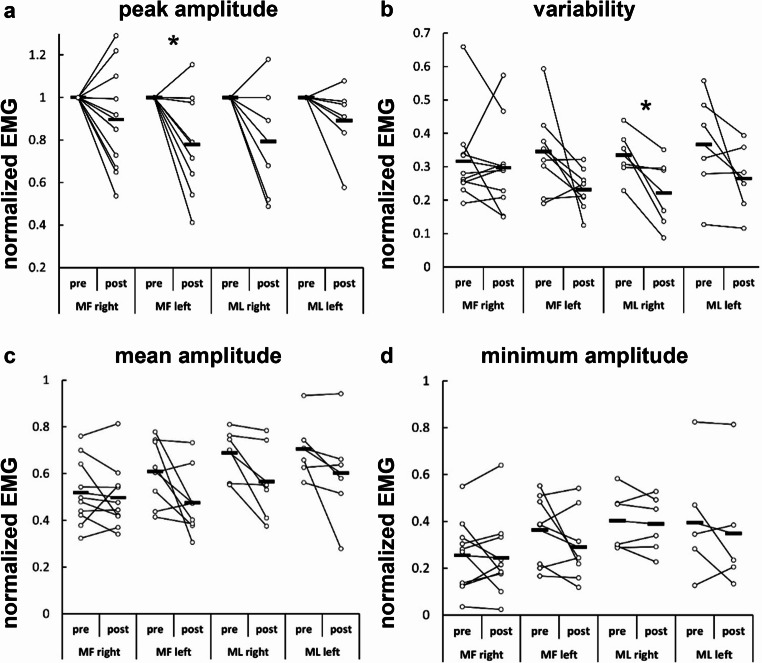
Normalized EMG outcomes pre- and post- hypertonic saline injection. **(a)** peak amplitude, **(b)** variability, **(c)** mean amplitude, **(d)** minimum amplitude. Hollow circles represent individual data of each rat, black bars represents the group mean. MF, multifidus muscle, ML, medial longissimus muscle; *p < 0.05, significant difference

After hypertonic saline injection, normalized peak amplitude significantly decreased in the left MF (*p* = 0.041, t = 2.50, effect size = 0.88, Fig. 5a), EMG variability decreased in the right ML (*p* = 0.030, t = 3.00, effect size = 1.23, Fig. 5b). No effects of nociception were detected in other EMG outcomes (minimum amplitude, mean amplitude) or muscles (right MF, left ML) (*p > 0.05*, Tables 2 and 3; Fig. 5c-d).

**Table 3:** Normalized EMG outcomes pre- and post- hypertonic saline injection

	MF right		MF left		^#^ML right		^#^ML left	
sample size	10		8		6		6	
	pre	post	pre	post	pre	post	pre	post
peak amplitude	/	0.90 (0.25)	/	0.78 (0.25)	/	0.79 (0.25)	/	0.89 (0.16)
minimum amplitude	0.25 (0.15)	0.24 (0.17)	0.36 (0.15)	0.29 (0.15)	0.40 (0.11)	0.39 (0.11)	0.39 (0.22)	0.35 (0.22)
mean amplitude	0.52 (0.14)	0.50 (0.14)	0.61 (0.14)	0.47 (0.14)	0.69 (0.10)	0.57 (0.15)	0.71 (0.12)	0.60 (0.20)
variability	0.32 (0.13)	0.3 (0.13)	0.35 (0.13)	0.23 (0.06)	0.33 (0.07)	0.22 (0.10)	0.37 (0.16)	0.26 (0.10)

MF, multifidus muscle; ML, medial longissimus muscle; ^#^data from ML dominant pattern. Peak EMG amplitude post-hypertonic saline injection was normalized to the peak value pre-hypertonic saline injection. Data are presented as mean (SD)

## Discussion

This study investigated the effects of experimentally induced nociception on the motor control of back muscles during locomotion in the rat. The main findings indicate that experimentally induced nociception did not affect spine and pelvis kinematics, as evidenced by consistent movement patterns and the lack of changes in gait parameters after hypertonic saline injection. In line with the kinematic consistency, the overall effects of nociception on EMG was also limited. The only significant effects were the decrease in the normalized peak amplitude of the left MF and the variability of the right ML. The overall EMG activation patterns and intermuscular coordination remained unchanged. These results suggest that the changes in EMG activity reflect a localized neuromuscular response to nociception rather than broader alterations in locomotion.

### Kinematic consistency during nociception

The lack of changes in kinematic variables indicates that acute nociception did not disrupt the locomotion pattern of the rats. There was a 31% increase in spine angle change after hypertonic saline injection, but this increase was within one degree (0.92 degree) and without statistical significance. These findings align with prior human studies, where hypertonic saline injection into the lumbar erector spinae muscle did not affect gait cycle duration (Arendt-Nielsen et al. 1996) or trunk kinematics (Lamoth et al. 2004). Nor were there any differences found in the amplitude of motion in the trunk, lumbar spine, pelvis, and hip during walking between people with LBP and healthy controls (Smith et al. 2022).

### Neuromuscular responses to nociception

The activation patterns of MF and ML muscles observed in this study are consistent with previous findings in rats and cats during locomotion (Geisler et al. 1996; Gramsbergen et al. 1999; Wada et al. 2006). Both left and right MF were active during the stride cycle, and there was simultaneous activation of bilateral MF with two peaks in each stride cycle (Geisler et al. 1996; Wada et al. 2006). ML activation occurred primarily during ipsilateral hind paw swing phase and the activity decreases to a tonic background level during the stance phase (Geisler et al. 1996). At adult age, both the MF and ML are tonically active most of the time when the rats are awake, and this is more pronounced in MF than ML (Geisler et al. 1996), which aligns with our findings that there were no actual rest periods in MF and ML throughout the stride cycle. MF and ML muscles in quadrupeds not only stabilize the trunk by counteracting lateral motion induced by limb movement, reducing overall spine movement, but also produced inward movement to algin the spine from the most lateral position to the midline during locomotion, especially ML activation showed a strong correlation to vertebral column movements (Wada et al. 2006). These findings align with our results, where MF demonstrated synchronized bilateral activation, suggesting its predominant role in spine stabilization, while ML exhibited alternating activation during the ipsilateral hind paw swing phase, contributing to lateral movement of the spine.

Despite the overall consistency of EMG activation patterns, the normalized peak amplitude of the left MF decreased by 22% and showed a general downward trend throughout the stride cycle. Although right MF and ML activation showed a similar downward trend, this was not statistically significant. Additionally, significant decrease in EMG variability following hypertonic saline injection was only found in the right ML. The general reduction in muscle activity was in contrast with previous findings of increased mean EMG activity during pain in humans (Arendt-Nielsen et al. 1996; Lamoth et al. 2004). Previous research has shown that experimentally induced muscle pain can reduce EMG activity in both painful and agonistic muscles during walking in humans (Graven-Nielsen et al. 1997). However, we only found reduction in EMG peak amplitude in one out of the four back muscles assessed. This may be unilateral pain leading to EMG changes ipsilateral to the site of pain induction only (Arendt-Nielsen et al. 1996). Our results suggest localized neuromuscular responses, consistent with reports of MF activity reduction in clinical LBP studies (Devecchi et al. 2023), where nociception (pain) inhibits the deep muscles. And the decreased variability in the right ML indicates a neuromuscular response aimed at stabilizing movement in response to nociceptive input.

The minimal changes in EMG are in agreement with the consistency of the kinematics, indicating that the neuromuscular responses were insufficient to alter locomotion mechanics. Hypertonic saline induced muscle nociceptive activity could modulate different levels of the motor pathways, inhibiting both the primary motor area and the spinal motoneurons (Le Pera et al. 2001). The EMG peak amplitude reduction in the left MF observed in the present study maybe related to central inhibition. Hypertonic saline induced pain does not impair neuromuscular transmission or muscle fibre conduction velocity (Farina et al. 2004; Qerama et al. 2005), but causes a significant decrease in firing rate of active motor units (Farina et al. 2004). A human study investigating corticomotor responses to acute LBP in humans demonstrated muscle-specific changes in motor evoked potentials, with some trunk muscles showing reduced excitability and others increased excitability (Tsao et al. 2011). These findings suggest that acute LBP can differentially influence descending corticomotor inputs to trunk muscles, likely based on their functional roles in spinal control, movement coordination, or their relation to the pain site.

This study has several limitations. Firstly, motor unit firing rates have been reported to be inversely correlated with pain intensity (Farina et al. 2004), the exact intensity of pain experienced by the rats after hypertonic saline injection remains unknown, although it was estimated as mild-to-moderate based on the Rat Grimace Scale. Secondly, our pilot experiment showed that the injection of 100 µl hypertonic saline is sufficient to elicit moderate nociceptive responses for at least three minutes. This volume may have been insufficient to induce long-lasting nociceptive signaling from the lumbar muscles of the rats, as previous studies showed that the hypertonic saline induced nociception lasts only for a few minutes (Paintal 1960; Taguchi et al. 2008). In addition, we did not record the specific side of the hypertonic saline injection (left or right MF), which limits our ability to determine the side-specific effects. However, our findings on peak amplitude indicate that there was bilateral general downward trend after hypertonic saline injection regardless of injection side. Lastly, only back muscles were assessed in this study, abdominal muscles were not examined. It would be beneficial to assess both back and abdominal muscles to obtain a more comprehensive understanding of how nociception influences overall trunk neuromuscular control.

In conclusion, our findings demonstrate that hypertonic saline injection induced nociception had minimal impact on motor control, with overall consistent kinematic and muscle activation patterns except for a localized decrease in peak EMG amplitude and variability. These findings highlight the response of the neuromuscular system in maintaining the functional movement under nociceptive conditions, and emphasize the importance of understanding how nociception affects motor control in a muscle-specific manner.

## Data Availability

Data and program codes used in analysis are available to researchers for academic purpose per request.
